# Current prevalence, changes, and determinants of breastfeeding practice in China: data from cross-sectional national household health services surveys in 2013 and 2018

**DOI:** 10.1186/s13006-023-00572-2

**Published:** 2023-08-11

**Authors:** Zeyu Li, Yufei Jia, Iris Parshley, Yaoguang Zhang, Jia Wang, Qian Long

**Affiliations:** 1 Global Health Research Center, Duke Kunshan University, Jiangsu, China; 2 Centre for Health Statistics and Information, National Health Commission, 38 Beilishi Road, Xicheng, Beijing, 100810 NO China; 3 Yuzhong District Center for Diseases Prevention and Control, 254 Heping Road, Yuzhong District, Chongqing, 400010 China

**Keywords:** Breastfeeding, China, Prevalence, Determinants, Motherhood, Maternity

## Abstract

**Background:**

The World Health Organization and the government of China have made many efforts to improve breastfeeding practices. The evidence of breastfeeding practices over the past decade in China is limited. The current study aimed to describe the current prevalence, variation trends, and determinants of breastfeeding practices in China using data from the National Household Health Service Surveys (NHHSS) in 2013 and 2018.

**Methods:**

Women who had at least one live birth in the five years from the 2013 NHHSS numbered 10,544, and 12,766 women from the 2018 NHHSS were included in the current study. The rates of breastfeeding, early initiation of breastfeeding within the first hour after birth, exclusive breastfeeding for at least six months since birth, and continued breastfeeding accompanied by adequate complementary feeding for over two years were measured. Logistic regressions were performed to study the associations between breastfeeding practices and maternal-based, healthcare-based, and infant-based characteristics.

**Results:**

In the 2018 survey, the rates of practiced any breastfeeding, early initiation of breastfeeding within the first hour after birth, exclusive breastfeeding for at least six months, and continued breastfeeding for over two years were 91.50%, 28.16%, 47.90%, and 4.78%, respectively, showing significant improvements compared to the 2013 survey period. Women who received high education, were from a household with high incomes, had more than one child, and had more antenatal and postnatal visits, were more likely to practice breastfeeding and initiate it within the first hour, but they were less likely to breastfeed the infants for two years. Births by caesarean section and low birthweight were associated with worse breastfeeding practices.

**Conclusions:**

The rates of practicing breastfeeding and exclusive breastfeeding for six months or more in China improved over the past decades, suggesting improved awareness and knowledge of breastfeeding among women. However, individual and social factors may impact practices of early initiation and continued breastfeeding. Strengthening breastfeeding support from family, community, and health professionals (e.g., family member engagement, friendly work environment, and professional consultation, etc.) during the postpartum and infant period may improve women’s confidence in breastfeeding practices.

**Supplementary Information:**

The online version contains supplementary material available at 10.1186/s13006-023-00572-2.

## Background

World Health Organization (WHO) recommends that all mothers initiate breastfeeding as soon as possible after delivery [1]. Beginning within the first hour has the greatest positive impact. The WHO also recommends exclusive breastfeeding for at least six months since birth, followed by continued breastfeeding with adequate complementary feeding for up to two years or beyond [1]. The Guidelines of Breastfeeding Promotion Strategies of China (2018) are accordant with the WHO recommendations and suggests the initiation of breastfeeding within the first 30 min after birth [2].

Globally, three in five neonates are not breastfed in the first hour of life, nearly two in three infants are not exclusively breastfed for at least six months, and only 45.1% are continually breastfed until two years old [3–5]. According to the Chinese National Nutrition and Health Survey, the crude rate of exclusive breastfeeding under six months in China declined from 27.6% in 2008 to 20.7% in 2013, and the sampling-weighted rate of exclusive breastfeeding was only 18.6% [6]. In addition, the Under-5 Child Nutrition and Health Surveillance System showed that the prevalence of initiation of breastfeeding within the first hour after birth was 55.84% and the prevalence of exclusive breastfeeding for at least six months was 34.90% in 2018 [5].

Many factors lead to poor breastfeeding practices around the world. A cross-sectional study in 16 sub-Saharan countries reported that mothers who completed primary school were more likely to breastfeed as recommended, but mothers with higher education did not show significantly different practices compared to mothers without education [7]. Breastfeeding stress, breastfeeding shame, physical pain and unfriendly social environment also hinder breastfeeding [8–10]. In China, some mothers believed that not initiating breastfeeding was better than interrupting and weaning, so they preferred to use infant formula milk and other alternative feeding strategies [11].

There is little information on the prevalence of breastfeeding in more recent decade in China. We performed a secondary data analysis using the National Household Health Service Surveys (NHHSS) of China in 2013 and 2018. Our study analyzed the current prevalence and variation trends in breastfeeding practices in China considering the rates of breastfeeding, early initiation of breastfeeding, exclusive breastfeeding (EBF), and continued breastfeeding (CBF) measures. Additionally, we studied the maternal-based, healthcare-based, and infant-based determinants of breastfeeding practices in China.

## Methods

### Data source and participants

The current study was based on the data from the NHHSS of China conducted in 2013 and 2018. Similar three-stage, stratified, cluster random sampling procedures were performed on both surveys. First, cities and counties were classified into five (in the 2018 survey) or six (in the 2013 survey) groups by socioeconomic, educational, demographic, and health status characteristics. The survey selected 90 cities and counties from each group with stratified sampling procedures, which were repeated six times to select ones with parameters (e.g., fertility rate, mortality rate, demographic structure, etc.) closest to the general population. Second, five sub-districts or townships were selected from each city and county according to local population size or per capita income. In the third stage, two communities or villages were randomly selected from each sub-district or township, and 60 households were randomly selected from each community or village.

Face-to-face surveys were conducted with all subjects from included households using a structured questionnaire by trained primary healthcare workers. Questionnaires used in the two surveys had the same structure and consisted of similar questions regarding the general demographic and socioeconomic characteristics and the utilization of health services. Information about the use of health services from 2008 to 2013 was collected in the 2013 survey, and that from 2014 to 2018 was collected in the 2018 survey. One section for women who had a live birth in the past five years involved questions about breastfeeding practices.

The participants of the study included the women who had at least one live birth in the five years in each round of the survey and had completed information on the date of child birth and infant status. This study included 10,544 women from the 2013 survey and 12,766 women from the 2018 survey.

### Measures

We assessed four breastfeeding measures in the 2018 survey including practiced any breastfeeding, initiation of breastfeeding within the first hour after birth, exclusive breastfeeding for six months or more, and continued breastfeeding for two years or more. Only two of the four outcomes (practiced any breastfeeding and exclusive breastfeeding for six months or more) were investigated in the 2013 survey. According to the WHO guidelines [1], the four outcome measures were defined as follows: practiced any breastfeeding indicated the child was breastfed at least once after birth; early initiation of breastfeeding represented the child had breastmilk for the first time or even tried sucking nipples within the first hour after birth; exclusive breastfeeding (EBF) signified the child was fed only with breastmilk and no other supplemental solid foods or liquids, including water except medications, vitamins, and minerals, from birth to six months or further [12]; continued breastfeeding (CBF) meant the child continued being fed with breastmilk, accompanied by adequate complementary feeding for two years or beyond. The questions were asked in each round survey: 1) Was your infant breastfed at least once after birth? (a) Yes; (b) No. 2) The time that your infant had breastmilk (including trying sucking nipples) for the first time: (a) ≥ 30 min after birth; (b) 30 min—60 min after birth; (c) 1 h—24 h after birth; (d) After the first 24 h after birth. 4) How many months were your infant exclusively breastfed? 5) How many months were your infant breastfed?

We examined maternal-based, healthcare-based, and infant-based characteristics associated with breastfeeding practices. Maternal-based characteristics consisted of 1) mother’s age at delivery (< 25 years, 25–34 years, ≥ 35 years); 2) mother’s educational level (illiterate or primary school, secondary school, high school or higher); 3) location of residence (urban, rural); 4) ethnicity (Han or other); 5) parity (1, ≥ 2); and 6) annual household income (divided into four quartiles with quartile 1 representing the lowest income group and quartile 4 representing the highest, CNY), which was a measure of savings and household expenditure during the calendar year preceding the survey (2012 for the survey in 2013, 2017 for the survey in 2018). The annual household income was converted to USD according to the exchange rate in 2022. Healthcare-based characteristics included 1) frequency of antenatal visits (< 5, 5–7, ≥ 8), defined as a clinical visit during pregnancy, except for the checkup before labor on the day of delivery; 2) mode of delivery (vaginal delivery or caesarean section); and 3) frequency of postnatal visits (0, ≥ 1), defined as postpartum medical tests or postpartum health consultation for mother and infant including breastfeeding guidance. The frequency of postnatal visits included all medical contacts up to 42 days after birth in the 2013 survey, but only up to 28 days after birth in the 2018 survey. Infant-based characteristics consist of 1) infant’s sex (male, female); and 2) infant’s birthweight (< 2500 g, 2500–4000 g, > 4000 g).

### Data analysis

Percentage, mean, and standard deviation were used to describe the maternal-based, healthcare-based, and infant-based characteristics of the participants. We performed Mann–Whitney U tests for continuous variables and chi-square tests for categorical variables to examine the differences in maternal-based, healthcare-based, and infant-based characteristics of participants between the two surveys. Outcome measures of breastfeeding practices were transformed into dichotomous variables and percentages were used to describe them. Chi-square tests were performed to investigate changes of breastfeeding practices across different groups divided by maternal-based, healthcare-based, and infant-based characteristics and survey year. Bivariate and multivariate logistic regressions were conducted to study the association between explanatory variables and breastfeeding practice, including the year of survey, maternal-based, healthcare-based, and infant-based characteristics. We also conducted logistic regressions in the databases of 2013 and 2018, respectively.

### Ethical statement, patient and public involvement

This study was based on a secondary data analysis. We obtained approval from the Center for Health Statistics and Information of the National Health Commission (NHC) of China (formerly the Ministry of Health) to access the NHHSS data in 2013 and 2018. The research team proposed the analysis plan and Y. Z, one of the co-authors, performed statistical analysis using STATA version 16.0 (Stata Corporation, USA). No patients were directly involved in the current study.

## Results

### Demographic and socioeconomic characteristics

From the 2013 survey, 10,544 women and 12,766 women from the 2018 survey were included in the current study. Their maternal-based, healthcare-based, and infant-based characteristics are shown in Table 1. The mothers’ age at delivery increased from 27.80 ± 5.35 years in the 2013 survey to 29.25 ± 5.24 years in the 2018 survey (*P* < 0.01). The proportion of women with the education of high school or higher level increased from 35.91% to 51.83% (*P* < 0.01). The ratio of women living in urban areas compared to those living in rural areas flipped between the two surveys, with more women living in cities in 2018 (*P* < 0.01). Forty-three-point fifty-two percent of women had two or more children in 2013, while 58.40% of women did in 2018 (*P* < 0.01). Annual household income increased significantly from 7953.90 USD to 11,212.84 USD (*P* < 0.01). The frequency of antenatal visits increased from 6.37 ± 3.71 times to 8.95 ± 4.41 times (*P* < 0.01). The proportion of women who had 8 or more antenatal visits was only 33.28% in 2013 but increased to 62.40% in 2018 (*P* < 0.01). The 2013 survey showed that 64.51% of women had at least one postnatal visit within 42 days after delivery, and 74.17% of women had a postnatal visit within 28 days after delivery in the 2018 survey (*P* < 0.01). As for the birthweight of infants, more children were reported as macrosomia (8.31%) in 2018 compared to 2013 (5.69%) (*P* < 0.01).

**Table 1:** Summary of the demographic and socioeconomic characteristics of participants. (Data from 2008 to 2018)

Characteristics	2008–2013 (*N* = 10,544)		2014–2018 (*N* = 12,766)		*P*
	N / Mean	% / SD	N / Mean	% / SD	
Maternal-based characteristics					
**Age at delivery (year)**	27.80	5.35	29.25	5.24	**< 0.01**
< 25	3184	30.20	2278	17.84	
25–34	6037	57.26	8349	65.40	
≥ 35	1322	12.54	2139	16.76	**< 0.01**
**Educational level**					
Illiterate or primary school	1755	16.64	1519	11.90	
Secondary school	5003	47.45	4630	36.27	
High school or higher	3786	35.91	6617	51.83	**< 0.01**
**Residence**					
Urban	4909	46.56	7087	55.51	
Rural	5635	53.44	5679	44.49	**< 0.01**
**Ethnicity**					
Han	9082	86.18	11,101	86.96	
Other	1457	13.82	1665	13.04	0.08
**Parity**	1.51	0.65	1.70	0.72	**< 0.01**
1	5955	56.48	5311	41.60	
≥ 2	4589	43.52	7455	58.40	**< 0.01**
**Annual household income**	53,561.64	215,601.50	75,507.31	160,776.40	**< 0.01**
Healthcare-based characteristics					
**Frequency of antenatal visit**	6.37	3.71	8.95	4.41	**< 0.01**
< 5	3182	30.21	1431	12.14	
5–7	3847	36.51	3003	25.47	
≥ 8	3506	33.28	7358	62.40	**< 0.01**
**Mode of delivery**					
Vaginal delivery	6149	58.37	6503	55.08	
Caesarean section	4385	41.63	5304	44.92	**< 0.01**
**Frequency of postnatal visit**^**a**^	1.27	1.34	1.26	1.11	**< 0.01**
0	3695	35.49	3047	25.83	
≥ 1	6715	64.51	8750	74.17	**< 0.01**
Infant-based characteristics					
**Sex**					
Male	5841	55.55	6355	53.82	
Female	4674	44.45	5452	46.18	**0.01**
**Birth weight (g)**	3288.23	692.33	3375.29	839.74	**< 0.01**
< 2500	464	4.43	544	4.61	
2500 ~ 4000	9411	89.88	10,273	87.08	
> 4000	596	5.69	980	8.31	**< 0.01**

*Abbreviations*: *N* Frequency, *SD* Standard deviation

^a^Frequency of postnatal visit was recorded within 42 days after birth in the 2013 survey, and in 28 days after birth in the 2018 survey

### Nationwide rates of breastfeeding and changes over time

The overall rate of breastfeeding increased from 86.95% in the 2013 survey to 91.50% in the 2018 survey, and the rate of EBF increased from 36.69% to 47.90% (Supplement Table 1). Significant improvement of breastfeeding practices was observed in almost every comparison pair (*P* < 0.05). The nationwide rate of early initiation was 28.16%, and the rate of CBF was only 4.78% in 2018 (Table 2). After adjusting for all explanatory variables, infants in the 2018 survey were more likely to receive breastmilk at least once (OR 1.45; 95% CI 1.31, 1.60, *P* < 0.01) and to be exclusively breastfed for six months or more (OR 1.83; 95% CI 1.71, 1.96, *P* < 0.01) compared to those in the 2013 survey (Table 3).

**Table 2:** Distribution of breastfeeding measures and demographic and socioeconomic characteristics. (Data from 2014 to 2018)

Characteristics	Practiced any breastfeeding			Early initiation of breastfeeding (EIBF)			Exclusive breastfeeding (EBF)			Continued breastfeeding (CBF)		
	Breastfed at least once		Total	Within the first hour		Total	For 6 months or more		Total	For 2 years or more		Total
	*N*	%	*N*	*N*	%	*N*	*N*	%	*N*	*N*	%	*N*
**Total**	9846	91.50	10,761	2773	28.16	9847	4400	47.90	9186	375	4.78	7842
Maternal-based characteristics												
**Age at delivery**												
< 25	1694	91.17	1858	471	27.80	1694	742	47.02	1578	54	3.88	1390
25–34	6522	91.87	7099	1872	28.70	6523	2958	48.69	6075	244	4.71	5178
≥ 35	1630	90.35	1804	430	26.38	1630	700	45.66	1533	77	6.04	1274
*P*	0.10		–	0.17		–	0.08		–	**0.03**		–
**Educational level**												
Illiterate or primary school	1108	88.29	1255	272	24.55	1108	493	46.91	1051	92	9.74	945
Secondary school	3565	90.57	3936	857	24.04	3565	1652	49.30	3351	129	4.45	2899
High school or higher	5173	92.87	5570	1644	31.77	5174	2255	47.14	4784	154	3.85	3998
*P*	**< 0.01**			**< 0.01**		–	0.12		–	**< 0.01**		–
**Residence**												
Urban	4374	91.24	4794	1003	22.93	4375	1995	48.78	4090	214	6.00	3569
Rural	5472	91.70	5967	1770	32.35	5472	2405	47.19	5096	161	3.77	4273
*P*	0.41		–	**< 0.01**		–	0.14		–	**< 0.01**		–
**Ethnicity**												
Han	8629	91.34	9447	2422	28.06	8630	4017	49.98	8038	289	4.24	6820
Other	1217	92.62	1314	351	28.84	1217	383	33.36	1148	86	8.41	1022
*P*	0.13		–	0.60		–	**< 0.01**		–	**< 0.01**		–
**Parity**												
1	3909	90.63	4313	1118	28.59	3910	1751	48.26	3628	128	4.16	3074
≥ 2	5937	92.08	6448	1655	27.88	5937	2649	47.66	5558	247	5.18	
*P*	**0.01**		–	0.45		–	0.59		–	**0.04**		–
**Household income quartiles**												
Quartile 1	2503	90.30	2772	612	24.45	2503	1106	47.29	2339	150	7.39	2029
Quartile 2	2322	91.24	2545	565	24.33	2322	1092	50.44	2165	98	5.31	1847
Quartile 3	3408	92.11	3700	1016	29.80	3409	1513	47.53	3183	89	3.29	2702
Quartile 4	1599	92.43	1730	577	36.09	1599	682	45.93	1485	38	3.04	1251
*P*	**0.03**		–	**< 0.01**		–	**0.04**		–	**< 0.01**		–
Healthcare-based characteristics												
**Frequency of antenatal visit**												
< 5	982	87.84	1118	239	24.34	982	435	46.38	938	75	8.87	
5 ~ 7	2287	91.01	2513	582	25.45	2287	1110	51.77	2144	120	6.34	
≥ 8	5898	92.77	6538	1788	30.31	5899	2505	46.13	5430	139	3.14	
*P*	**< 0.01**		–	**< 0.01**		–	**< 0.01**		–	**< 0.01**		–
**Mode of delivery**												
Vaginal delivery	5063	92.90	5450	1704	33.66	5063	2268	48.45	4681	46	4.92	935
Caesarean section	4111	90.37	4549	905	22.01	4112	1784	46.48	3838	6	11.32	53
*P*	**< 0.01**		–	**< 0.01**		–	0.07		–	0.09		–
**Frequency of postnatal visit**												
0	2223	89.42	2486	496	22.31	2223	1017	48.15	2112	105	5.64	1863
≥ 1	6948	92.54	7508	2113	30.41	6949	3034	47.37	6405	230	4.33	5313
*P*	**< 0.01**		–	**< 0.01**		–	0.55		–	**0.03**		–
Infant-based characteristics												
**Sex**												
Male	4969	91.70	5419	1400	28.17	4970	2221	47.98	4629	189	4.83	3914
Female	4205	91.81	4580	1209	28.75	4205	1831	47.07	3890	146	4.47	3264
*P*	0.86		–	0.55		–	0.41		–	0.51		–
**Birth weight**												
< 2500	374	83.11	450	99	26.47	374	135	38.14	354	22	7.24	304
2500 ~ 4000	8035	92.23	8712	2332	29.02	8036	3544	47.51	7460	278	4.44	6265
> 4000	759	91.45	830	178	23.45	759	370	52.86	700	35	5.79	604
*P*	**< 0.01**		–	**< 0.01**		–	**< 0.01**		–	**0.03**		–

*Abbreviations*: *N* Frequency

**Table 3:** Logistic regressions of breastfeeding measures and demographic and socioeconomic characteristics. (Data from 2008 to 2018)

Characteristics	Practiced any breastfeeding				Exclusive breastfeeding for 6 months or more (EBF)			
	OR	95%CI		*P*	OR	95%CI		*P*
**Year**								
2008–2013 (Ref.)	1.00				1.00			
2014–2018	**1.45**	**1.31**	**1.60**	** < 0.01**	**1.83**	**1.71**	**1.96**	** < 0.01**
Maternal-based characteristics								
**Age at delivery (year)**								
< 25 (Ref.)	1.00				1.00			
25–34	1.04	0.92	1.16	0.55	1.00	0.92	1.08	0.99
≥ 35	0.89	0.75	1.04	0.15	0.90	0.80	1.01	0.08
**Educational level**								
Illiterate or primary school (Ref.)	1.00				1.00			
Secondary school	**1.18**	**1.03**	**1.35**	**0.02**	1.00	0.91	1.10	0.96
High school or higher	**1.40**	**1.19**	**1.63**	** < 0.01**	0.93	0.83	1.04	0.22
**Residence**								
Urban (Ref.)	1.00				1.00			
Rural	1.21	1.10	1.35	< 0.01	1.12	1.05	1.20	< 0.01
Ethnicity								
Han (Ref.)	1.00				1.00			
The other	**1.31**	**1.12**	**1.52**	** < 0.01**	**0.54**	**0.49**	**0.59**	** < 0.01**
**Parity**								
1 (Ref.)	1.00				1.00			
≥ 2	**1.33**	**1.19**	**1.48**	** < 0.01**	1.03	0.96	1.11	0.43
**Household income quartiles**								
Quartile 1(Ref.)	1.00				1.00			
Quartile 2	1.01	0.90	1.15	0.83	**1.10**	**1.01**	**1.19**	**0.04**
Quartile 3	1.02	0.90	1.16	0.78	0.98	0.90	1.07	0.66
Quartile 4	0.99	0.86	1.15	0.95	0.97	0.88	1.08	0.63
Healthcare-based characteristics								
**Frequency of antenatal visit**								
< 5 (Ref.)	1.00				1.00			
5 ~ 7	1.12	0.99	1.27	**0.07**	**0.82**	**0.75**	**0.90**	** < 0.01**
≥ 8	**1.38**	**1.21**	**1.57**	** < 0.01**	**0.61**	**0.56**	**0.67**	** < 0.01**
**Mode of delivery**								
Vaginal delivery (Ref.)	1.00				1.00			
Caesarean section	**0.70**	**0.64**	**0.77**	** < 0.01**	0.98	0.92	1.05	0.63
**Frequency of postnatal visit in the first 42 days**								
0 (Ref.)	1.00				1.00			
≥ 1	**1.20**	**1.09**	**1.32**	** < 0.01**	0.97	0.91	1.04	0.45
Infant-based characteristics								
**Sex**								
Male (Ref.)	1.00				1.00			
Female	1.00	0.91	1.09	0.94	1.02	0.96	1.09	0.50
**Birth weight (g)**								
< 2500 (Ref.)	1.00				1.00			
2500 ~ 4000	**2.06**	**1.73**	**2.46**	** < 0.01**	**1.32**	**1.13**	**1.55**	** < 0.01**
> 4000	**2.10**	**1.65**	**2.68**	** < 0.01**	**1.50**	**1.24**	**1.83**	** < 0.01**

*Abbreviations*: *OR* Odds ratio, *CI* Confidence interval

### Factors associated with breastfeeding practices in 2014–2018

Table 2 presents the breastfeeding behaviors grouped by maternal-based, healthcare-based, and infant-based characteristics between 2014 and 2018 (data from the 2018 survey). Table 4 shows the associations between breastfeeding practice and the characteristics. The overall rate of breastfeeding was higher for mothers with higher educational level (*P* < 0.01), greater parity (*P* < 0.01), more antenatal and postnatal visits (*P* < 0.01), vaginal delivery (*P* < 0.01), and normal infant birthweight (*P* < 0.01) (Table 2). All these variables remained significantly associated with breastfeeding practice after adjusting for all explanatory variables; however, we also found that mothers from ethnic minorities were more likely to practice breastfeeding (OR 1.34; 95% CI 1.05, 1.71, *P* = 0.02) than Han women (Table 4).

**Table 4:** Logistic regressions of breastfeeding measures and demographic and socioeconomic characteristics. (Data from 2014 to 2018)

Characteristics	Practiced any breastfeeding				Early initiation of breastfeeding within the first hour after birth (EIBF)				Exclusive breastfeeding for 6 months or more (EBF)				Continued breastfeeding for 2 years or more (CBF)			
	OR	95%CI		*P*	OR	95%CI		*P*	OR	95%CI		*P*	OR	95%CI		*P*
Maternal-based characteristics																
**Age at delivery (year)**																
< 25 (Ref.)	1.00				1.00				1.00				1.00			
25–34	0.92	0.75	1.13	0.44	0.93	0.81	1.06	0.29	1.10	0.98	1.25	0.12	**1.51**	**1.08**	**2.12**	**0.02**
≥ 35	0.87	0.67	1.14	0.32	**0.81**	**0.68**	**0.97**	**0.02**	0.98	0.83	1.15	0.78	**1.58**	**1.03**	**2.41**	**0.04**
**Educational level**																
Illiterate or primary school (Ref.)	1.00				1.00				1.00				1.00			
Secondary school	**1.28**	**1.02**	**1.61**	**0.03**	0.86	0.73	1.03	0.10	0.99	0.85	1.15	0.89	**0.58**	**0.42**	**0.79**	** < 0.01**
High school or higher	**1.96**	**1.52**	**2.52**	** < 0.01**	1.07	0.89	1.29	0.45	0.94	0.79	1.10	0.43	0.73	0.51	1.04	0.08
**Residence**																
Urban (Ref.)	1.00				1.00				1.00				1.00			
Rural	1.15	0.97	1.36	0.11	**0.64**	**0.57**	**0.72**	** < 0.01**	1.10	1.00	1.22	0.06	1.10	0.85	1.43	0.47
**Ethnicity**																
Han (Ref.)	1.00				1.00				1.00				1.00			
Other	**1.34**	**1.05**	**1.71**	**0.02**	1.14	0.98	1.32	0.09	**0.47**	**0.41**	**0.55**	** < 0.01**	**1.40**	**1.04**	**1.89**	**0.03**
**Parity**																
1 (Ref.)	1.00				1.00				1.00				1.00			
≥ 2	**1.47**	**1.24**	**1.73**	** < 0.01**	**1.24**	**1.12**	**1.39**	** < 0.01**	0.93	0.84	1.03	0.15	0.84	0.64	1.10	0.20
**Household income quartiles**																
Quartile 1 (Ref.)	1.00				1.00				1.00				1.00			
Quartile 2	1.08	0.88	1.32	0.45	0.97	0.84	1.11	0.63	1.11	0.98	1.26	0.10	0.85	0.64	1.13	0.26
Quartile 3	1.10	0.90	1.34	0.37	**1.15**	**1.00**	**1.31**	**0.05**	1.01	0.89	1.14	0.89	**0.54**	**0.39**	**0.74**	** < 0.01**
Quartile 4	1.04	0.81	1.35	0.76	**1.36**	**1.16**	**1.60**	** < 0.01**	0.98	0.84	1.14	0.78	**0.54**	**0.35**	**0.82**	**0.01**
Healthcare-based characteristics																
**Frequency of antenatal visit**																
< 5 (Ref.)	1.00				1.00				1.00				1.00			
5 ~ 7	**1.37**	**1.08**	**1.73**	**0.01**	1.03	0.86	1.23	0.74	1.12	0.96	1.31	0.16	0.82	0.60	1.12	0.22
≥ 8	**1.65**	**1.32**	**2.07**	** < 0.01**	1.09	0.92	1.29	0.32	0.88	0.75	1.02	0.10	**0.46**	**0.33**	**0.64**	** < 0.01**
**Mode of delivery**																
Vaginal delivery (Ref.)	1.00				1.00				1.00				1.00			
Caesarean section	**0.70**	**0.61**	**0.81**	** < 0.01**	**0.54**	**0.49**	**0.59**	** < 0.01**	**0.89**	**0.81**	**0.97**	**0.01**	1.06	0.84	1.33	0.64
**Frequency of postnatal visit**																
0(Ref.)	1.00				1.00				1.00				1.00			
≥ 1	**1.31**	**1.12**	**1.54**	** < 0.01**	**1.42**	**1.26**	**1.59**	** < 0.01**	0.99	0.90	1.10	0.89	0.90	0.71	1.15	0.42
Infant-based characteristics																
**Sex**																
Male (Ref.)	1.00				1.00				1.00				1.00			
Female	1.04	0.90	1.20	0.59	1.03	0.94	1.13	0.54	0.99	0.91	1.08	0.86	0.91	0.73	1.14	0.40
**Birth weight (g)**																
< 2500 (Ref.)	1.00				1.00				1.00				1.00			
2500 ~ 4000	**2.20**	**1.69**	**2.87**	** < 0.01**	1.08	0.85	1.38	0.52	**1.43**	**1.15**	**1.79**	** < 0.01**	0.67	0.42	1.07	0.09
> 4000	**2.17**	**1.53**	**3.09**	** < 0.01**	0.87	0.65	1.17	0.36	**1.77**	**1.36**	**2.31**	** < 0.01**	0.84	0.48	1.48	0.55

*Abbreviations*: *OR* Odds ratio, *CI* Confidence interval

As for exclusive breastfeeding, a higher percentage of mothers who were of Han ethnicity (*P* < 0.01), had children with over 4000 g birthweight (*P* < 0.01), and attended more antenatal visits (*P* < 0.01) practiced EBF (Table 2). After adjusting for all explanatory variables, the association between EBF and antenatal visits was not statistically significant. Additionally, we found women giving birth vaginally were more likely to practice EBF (OR 0.89; 95% CI 0.81, 0.97, *P* = 0.01) than women who had a caesarean section (Table 4).

Early initiation of breastfeeding was seen more in the women with higher educational level (*P* < 0.01), rural residency (*P* < 0.01), higher household income (*P* < 0.01), more antenatal and postnatal visits (*P* < 0.01), vaginal delivery (*P* < 0.01), and normal infant birthweight (*P* < 0.01) (Table 2). However, the logistic regression analysis found that maternal educational level, antenatal visits, and infant birthweight were not statistically associated with early initiation of breastfeeding. The highest household income category (OR 1.36; 95% CI 1.16, 1.60, *P* < 0.01), vaginal delivery (OR 0.54; 95% CI 0.49, 0.59, *P* < 0.01), and the frequency of the postnatal visits (OR 1.42; 95% CI 1.26, 1.59, *P* < 0.01) were statistically associated with early initiation of breastfeeding. Women who had two or more children (OR 1.24; 95% CI 1.12, 1.39, *P* < 0.01), were younger than 35 years (OR 0.81; 95% CI 0.68, 0.97, *P* = 0.02), and lived in urban areas (OR 0.64; 95% CI 0.57, 0.72, *P* < 0.01) were more likely to start breastfeeding within the first hour after birth (Table 4).

Regarding the length of breastfeeding, women who received secondary school education (OR 0.58; 95% CI 0.42, 0.79, *P* < 0.01), were from households with higher income (OR 0.54; 95% CI 0.35, 0.82, *P* = 0.01), and had more than eight antenatal visits (OR 0.46; 95% CI 0.33, 0.64, *P* < 0.01) were less likely to continue breastfeeding for over two years compared to their counterparts. However, women who were over 35 years at delivery (OR 1.58; 95% CI 1.03, 2.41, *P* = 0.04) or were in an ethnic minority (OR 1.40; 95% CI 1.04, 1.89, *P* = 0.03) were more likely to breastfeed for two years or more (Table 4).

We found similar factors associated with breastfeeding and exclusive breastfeeding in 2008–2013 (Supplement Tables 2 and 3). Data is not shown here.

## Discussion

We found that breastfeeding practices improved over time, particularly the increase of the rate of exclusive breastfeeding. The maternal and child health professional associations and breastfeeding campaigns actively promoted breastfeeding practices in China, especially exclusive breastfeeding for at least six months, through public education and guidance for health professionals. More recently, China’s Outlines for Children’s Development (2021–2030) introduced a goal of no less than 50% of exclusive breastfeeding by 2025. Nevertheless, the rates of early initiation of breastfeeding and continued breastfeeding remained low.

Consistent with other studies [13, 14], we found that women with more than one child practiced breastfeeding more, had an increased likelihood of early initiation of breastfeeding, and had higher rates of CBF compared to those with only one child. Women who have more children may gain better knowledge and have experience with breastfeeding, thus have better awareness and practice more breastfeeding. In addition, higher maternal educational levels and better household incomes were positively associated with practiced any breastfeeding and early initiation, but was negatively associated with continued breastfeeding. Women who were well educated may understand the importance of breastfeeding their infant, but they may need to return to work after maternity leave for 4–6 months, which may hinder continued breastfeeding due to time constraints and unfriendly breastfeeding environments at the workplace [15, 16]. Additionally, households with better incomes are willing and able to pay for breastmilk substitutes, which may lead to early cessation of breastfeeding [17].

Women with more antenatal and postnatal visits may gain better knowledge and professional support on breastfeeding, which were positively associated with breastfeeding practices, despite those were not significantly associated with EBF and continued breastfeeding. Consistent with previous studies in other countries [18, 19], birth by caesarean section negatively impacted breastfeeding practices. The previous studies reported that positioning difficulty, more pain and fatigue, and anesthesia residues for women who had caesarean section may lead to difficulty in breastfeeding practices [20, 21]. Early separation, interrupted lactation, and inhibited infant suckling may be mediating factors between caesarean section and delayed breastfeeding initiation [22]. Additionally, physiological causes related to either emergency or premature elective caesarean sections may decrease the likelihood of early initiation of breastfeeding [23]. It was also suggested that delayed initiation of breastfeeding related to caesarean sections could be prevented by a conscious, pro-breastfeeding, supportive healthcare environment [19].

Low birthweight was negatively associated with breastfeeding practices in this study, which was consistent with previous studies in other countries [24, 25]. Preterm birth, which is usually the cause of low birthweight, can be attributed to the mother’s immature lactation system, the infant’s inability to suck, and separation of mothers and infants after delivery [26]. Additionally, the previous studies reported that health professionals and families believed that breastmilk substitutes or other complementary food provided more nutrients for infants with low birthweight. This might be a possible reason for the absence of exclusive breastfeeding or early cessation of breastfeeding for low-birthweight babies.

To promote breastfeeding practices, supports from families, society, and health professionals are critical. Mental health issues among postnatal mothers, particularly depression, stress, and anxiety negatively affect infant care including breastfeeding practices [27, 28]. Previous studies also found that family members’ support increased the likelihood of better breastfeeding practices [11, 29]. Hence, involvement of family members in antenatal and postnatal care will contribute to appropriate breastfeeding practices. Additionally, when women return to work after maternity leave, a friendly work environment, such as having a breastfeeding room or allowing women to leave early in a workday may encourage continued breastfeeding [30, 31].

Yang et al. reported that many Chinese health professionals’ knowledge about breastfeeding was insufficient, and breastfeeding promotion and education during pregnancy and early postpartum periods were suboptimal [32]. Strengthening breastfeeding training for primary healthcare providers, midwives, and nurses who provide antenatal and postnatal health education and promotion will be critical for providing support for pregnant and postpartum women. In China, caesarean section rates increased rapidly over the past three decades and many caesarean sections are not medically indicated, which is caused by a complex of individual, social, cultural, and health system factors [33]. Thus, a comprehensive intervention to mitigate unnecessary caesarean section will also contribute to better breastfeeding practices.

This study used the most recent nationwide data on breastfeeding practice in China. However, there are several limitations of this study. First, the cross-sectional survey design limited the possibility to test the causality between breastfeeding practice and explanatory variables. Additionally, the participants suffered from recall bias. In this study, around one-third of women reported their breastfeeding practices in 4–5 years prior to the survey. Therefore, the reported timing of breastfeeding initiation and length of exclusive breastfeeding may not be accurate. Finally, maternal history and medical records were not available in the survey data. We were not able to explore breastfeeding practices by different women’s health statuses.

## Conclusions

Over the past decade, the rates of practicing breastfeeding and exclusive breastfeeding have significantly improved, suggesting improved awareness and knowledge of breastfeeding among women; however, the rates of early initiation of breastfeeding and continued breastfeeding remained low. Familial, societal, and healthcare professionals’ support is critical to encourage early and continued breastfeeding. Strengthening breastfeeding support from family, community, and health professionals (e.g., family member engagement, friendly work environment, and professional consultation, etc.) during the postpartum and infant period may improve women’s confidence in breastfeeding practices.

### Supplementary Information

**Additional file 1:Supplement Table 1.** Comparisons between the 2013 and 2018 surveys’ distribution of breastfeeding measures, and demographic and socioeconomic characteristics. **Supplement Table 2.** Distribution of breastfeeding measures and demographic and socioeconomic characteristics. (Data from 2008 to 2013). **Supplement Table 3.** Logistic regressions of breastfeeding measures and demographic and socioeconomic characteristics. (Data from 2008 to 2013).

## Data Availability

The datasets used during the current study are available from the corresponding author upon detailed request.

## Abbreviations

CBF: Continued breastfeeding
EBF: Exclusive breastfeeding
NHC: National Health Commission
NHHSS: The National Household Health Service Surveys
WHO: World Health Organization
