# Supplementary material for: Current prevalence, changes, and determinants of breastfeeding practice in China: data from cross-sectional national household health services surveys in 2013 and 2018
Source: Int Breastfeed J. 2023 Aug 11;18:40. doi: 10.1186/s13006-023-00572-2 (PMC10416475; doi:10.1186/s13006-023-00572-2)
Supplement: Supplementary file 1 — Additional file 1:Supplement Table 1. Comparisons between the 2013 and 2018 surveys’ distribution of breastfeeding measures, and demographic and socioeconomic characteristics. Supplement Table 2. Distribution of breastfeeding measures and demographic and socioeconomic characteristics. (Data from 2008 to 2013). Supplement Table 3. Logistic regressions of breastfeeding measures and demographic and socioeconomic characteristics. (Data from 2008 to 2013). [file 13006_2023_572_MOESM1_ESM.docx]

**Current prevalence, changes, and determinants of breastfeeding practice in china: data from cross-sectional National Household Health Services Surveys in 2013 and 2018**

Zeyu Li^1*^, Yufei Jia^1*^, Iris Parshley^1^, Yaoguang Zhang^2^, Jia Wang^3^, Qian Long^1^

^1^ Global Health Research Center, Duke Kunshan University, Jiangsu, China

^2^ Centre for Health Statistics and Information, National Health Commission, Beijing, China

^3^ Yuzhong district Center for Diseases Prevention and Control, Chongqing, China

*: Both authors contributed equally to the work.

**Co-correspondence to:**

Jia Wang

Yuzhong District Center for Disease Control and Prevention，

254 Heping Road, Yuzhong District, Chongqing 400010, China

Email: 20472080@qq.com

Phone: +86 18875273982

Yaoguang Zhang

Centre for Health Statistics and Information, National Health Commission

NO.38 Beilishi road, Xicheng, Beijing, China,100810

Email: [13910844351@163.com](mailto:13910844351@163.com)

Phone: +86 10 6879 2524

**Supplement Table 1**. Comparisons between the 2013 and 2018 surveys’ distribution of breastfeeding measures, and demographic and socioeconomic characteristics.

| Characteristics | Rate of practiced any breastfeeding (%) | | |  | Rate of exclusive breastfeeding for 6 months or more (EBF) (%) | | |
| --- | --- | --- | --- | --- | --- | --- | --- |
|  | 2008-2013 | 2014-2018 | *P* |  | 2008-2013 | 2014-2018 | *P* |
| **Total** | 86.95 | 91.50 | **< 0.01** |  | 36.69 | 47.90 | **< 0.01** |
| Maternal-based characteristics | | | | | | | |
| **Age at delivery (year)** |  |  |  |  |  |  |  |
| <25 | 86.27 | 91.17 | **< 0.01** |  | 38.16 | 47.02 | **< 0.01** |
| 25-34 | 87.78 | 91.87 | **< 0.01** |  | 35.75 | 48.69 | **< 0.01** |
| ≥35 | 84.78 | 90.35 | **< 0.01** |  | 37.48 | 45.66 | **< 0.01** |
| **Educational level** |  |  |  |  |  |  |  |
| Illiterate or primary school | 86.94 | 88.29 | 0.26 |  | 39.29 | 46.91 | **< 0.01** |
| Secondary school | 87.62 | 90.57 | **< 0.01** |  | 39.70 | 49.30 | **< 0.01** |
| High school or higher | 86.07 | 92.87 | **< 0.01** |  | 31.44 | 47.14 | **< 0.01** |
| **Residence** |  |  |  |  |  |  |  |
| Urban | 88.58 | 91.24 | **< 0.01** |  | 40.19 | 48.78 | **< 0.01** |
| Rural | 85.08 | 91.70 | **< 0.01** |  | 32.52 | 47.19 | **< 0.01** |
| **Ethnicity** |  |  |  |  |  |  |  |
| Han | 86.45 | 91.34 | **< 0.01** |  | 37.68 | 49.98 | **< 0.01** |
| Other | 90.11 | 92.62 | **0.02** |  | 30.72 | 33.36 | 0.17 |
| **Parity** |  |  |  |  |  |  |  |
| 1 | 85.95 | 90.63 | **< 0.01** |  | 33.95 | 48.26 | **< 0.01** |
| ≥2 | 88.25 | 93.61 | **< 0.01** |  | 40.15 | 39.80 | **< 0.01** |
| **Household income quartiles** |  |  |  |  |  |  |  |
| Quartile 1 | 87.95 | 90.30 | **< 0.01** |  | 39.42 | 47.29 | **< 0.01** |
| Quartile 2 | 86.86 | 91.24 | **< 0.01** |  | 39.55 | 50.44 | **< 0.01** |
| Quartile 3 | 86.30 | 92.11 | **< 0.01** |  | 34.07 | 47.53 | **< 0.01** |
| Quartile 4 | 86.60 | 92.43 | **< 0.01** |  | 32.54 | 45.93 | **< 0.01** |
| Healthcare-based characteristics | | | | | | | |
| **Frequency of antenatal visit** |  |  |  |  |  |  |  |
| ＜5 | 86.54 | 78.99 | 0.29 |  | 45.02 | 52.00 | 0.50 |
| 5~7 | 86.81 | 90.70 | **< 0.01** |  | 38.23 | 49.82 | **< 0.01** |
| ≥8 | 87.44 | 93.05 | **< 0.01** |  | 27.58 | 45.52 | **< 0.01** |
| **Mode of delivery** |  |  |  |  |  |  |  |
| Vaginal delivery | 88.79 | 92.90 | **< 0.01** |  | 36.49 | 48.45 | **< 0.01** |
| Caesarean section | 84.37 | 90.37 | **< 0.01** |  | 37.02 | 46.48 | **< 0.01** |
| **Frequency of postnatal visit** |  |  |  |  |  |  |  |
| 0 | 85.91 | 89.42 | **< 0.01** |  | 38.73 | 48.15 | **< 0.01** |
| ≥1 | 87.46 | 92.54 | **< 0.01** |  | 35.57 | 47.37 | **< 0.01** |
| Infant-based characteristics | | | | | | | |
| **Sex** |  |  |  |  |  |  |  |
| Male | 87.07 | 91.70 | **< 0.01** |  | 36.24 | 47.98 | **< 0.01** |
| Female | 86.75 | 91.81 | **< 0.01** |  | 37.25 | 47.07 | **< 0.01** |
| **Birth weight (g)** |  |  |  |  |  |  |  |
| ＜2500 | 77.59 | 83.11 | **0.05** |  | 33.61 | 38.14 | 0.29 |
| 2500~4000 | 87.36 | 92.23 | **< 0.01** |  | 36.78 | 47.51 | **< 0.01** |
| ＞4000 | 87.56 | 91.45 | **0.02** |  | 37.07 | 52.86 | **< 0.01** |

**Supplement Table 2.** Distribution of breastfeeding measures and demographic and socioeconomic characteristics. (Data from 2008 to 2013)

| Characteristics | Practiced any breastfeeding | | | |  | Exclusive breastfeeding (EBF) | | | |
| --- | --- | --- | --- | --- | --- | --- | --- | --- | --- |
|  | Breastfed at least once | | Total | |  | For 6 months or more | | Total |  |
|  | N | % | N |  | | N | % | N |  |
| **Total** | 9162 | 86.95 | 10537 |  | | 3351 | 36.69 | 9133 |  |
| Maternal-based characteristics | | | | | | | | |  |
| **Age at delivery** |  |  |  |  | |  |  |  |  |
| <25 | 2745 | 86.27 | 3182 |  | | 1043 | 38.16 | 2733 |  |
| 25-34 | 5296 | 87.78 | 6033 |  | | 1888 | 35.75 | 5281 |  |
| ≥35 | 1120 | 84.78 | 1321 |  | | 419 | 37.48 | 1118 |  |
| *P* | **0.01** | | -- | |  | 0.09 | | -- |  |
| **Educational level** |  |  |  |  | |  |  |  |  |
| Illiterate or primary school | 1525 | 86.94 | 1754 |  | | 596 | 39.29 | 1517 |  |
| Secondary school | 4380 | 87.62 | 4999 |  | | 1733 | 39.70 | 4365 |  |
| High school or higher | 3257 | 86.07 | 3784 |  | | 1022 | 31.44 | 3251 |  |
| *P* | 0.09 | | -- | |  | **< 0.01** | | -- |  |
| **Residence** |  |  |  |  | |  |  |  |  |
| Urban | 4998 | 88.58 | 5631 |  | | 1996 | 40.19 | 4966 |  |
| Rural | 4174 | 85.08 | 4906 |  | | 1355 | 32.52 | 4167 |  |
| *P* | **< 0.01** | | -- | |  | **< 0.01** | | -- |  |
| **Ethnicity** |  |  |  |  | |  |  |  |  |
| Han | 7846 | 86.45 | 9076 |  | | 2949 | 37.68 | 7827 |  |
| Other | 1312 | 90.11 | 1456 |  | | 400 | 30.72 | 1302 |  |
| *P* | **< 0.01** | | -- | |  | **< 0.01** | | -- |  |
| **Parity** |  |  |  |  | |  |  |  |  |
| 1 | 5115 | 85.95 | 5951 |  | | 1730 | 33.95 | 5096 |  |
| ≥2 | 4047 | 88.25 | 4586 |  | | 1621 | 40.15 | 4037 |  |
| *P* | **< 0.01** | | -- | |  | **< 0.01** | | -- |  |
| **Household income quartiles** |  |  |  |  | |  |  |  |  |
| Quartile 1 | 2364 | 87.95 | 2688 |  | | 928 | 39.42 | 2354 |  |
| Quartile 2 | 2657 | 86.86 | 3059 |  | | 1046 | 39.55 | 2645 |  |
| Quartile 3 | 2122 | 86.30 | 2459 |  | | 722 | 34.07 | 2119 |  |
| Quartile 4 | 2017 | 86.60 | 2329 |  | | 655 | 32.54 | 2013 |  |
| *P* | 0.39 | | -- | |  | **< 0.01** | | -- |  |
| Healthcare-based characteristics | | | | | | | | |  |
| **Frequency of antenatal visit** |  |  |  |  | |  |  |  |  |
| ＜5 | 2751 | 86.54 | 3179 |  | | 1234 | 45.02 | 2741 |  |
| 5~7 | 3338 | 86.81 | 3845 |  | | 1273 | 38.23 | 3330 |  |
| ≥8 | 3064 | 87.44 | 3504 |  | | 842 | 27.58 | 3053 |  |
| *P* | 0.52 | | -- | |  | **< 0.01** | | -- |  |
| **Mode of delivery** |  |  |  |  | |  |  |  |  |
| Vaginal delivery | 5456 | 88.79 | 6145 |  | | 1985 | 36.49 | 5440 |  |
| Caesarean section | 3697 | 84.37 | 4382 |  | | 1364 | 37.02 | 3684 |  |
| *P* | **< 0.01** | | -- | |  | 0.57 | | -- |  |
| **Frequency of postnatal visit** |  |  |  |  | |  |  |  |  |
| 0 | 3171 | 85.91 | 3691 |  | | 1223 | 38.73 | 3158 |  |
| ≥1 | 5870 | 87.46 | 6712 |  | | 2082 | 35.57 | 5854 |  |
| *P* | **0.02** | | -- | |  | **< 0.01** | | -- |  |
| Infant-based characteristics | | | | | | | | |  |
| **Sex** |  |  |  |  | |  |  |  |  |
| Male | 5082 | 87.07 | 5837 |  | | 1836 | 36.24 | 5066 |  |
| Female | 4052 | 86.75 | 4671 |  | | 1505 | 37.25 | 4040 |  |
| *P* | 0.61 | | -- | |  | 0.28 | | -- |  |
| **Birth weight** |  |  |  |  | |  |  |  |  |
| ＜2500 | 360 | 77.59 | 464 |  | | 121 | 33.61 | 360 |  |
| 2500~4000 | 8217 | 87.36 | 9406 |  | | 3013 | 36.78 | 8192 |  |
| ＞4000 | 521 | 87.56 | 594 |  | | 192 | 37.07 | 518 |  |
| *P* | **< 0.01** | | -- | |  | 0.57 | | -- |  |

Abbreviations: N-frequency.

**Supplement Table 3.** Logistic regressions of breastfeeding measures and demographic and socioeconomic characteristics. (Data from 2008 to 2013)

| **Characteristics** | Practiced any breastfeeding | | | |  | Exclusive breastfeeding for 6 months or more (EBF) | | | |
| --- | --- | --- | --- | --- | --- | --- | --- | --- | --- |
|  | OR | 95%CI | | *P* |  | OR | 95%CI | | *P* |
| Maternal-based characteristics | | | | | | | | | |
| **Age at delivery (year)** |  |  |  |  |  |  |  |  |  |
| <25 (Ref.) | 1.00 |  |  |  |  | 1.00 |  |  |  |
| 25-34 | 1.13 | 0.98 | 1.30 | 0.09 |  | 0.91 | 0.82 | 1.01 | 0.08 |
| ≥35 | 0.87 | 0.71 | 1.07 | 0.20 |  | **0.83** | **0.71** | **0.98** | **0.03** |
| **Educational level** |  |  |  |  |  |  |  |  |  |
| Illiterate or primary school (Ref.) | 1.00 |  |  |  |  | 1.00 |  |  |  |
| Secondary school | 1.12 | 0.94 | 1.33 | 0.20 |  | 1.04 | 0.92 | 1.18 | 0.54 |
| High school or higher | 1.10 | 0.90 | 1.34 | 0.37 |  | 0.93 | 0.80 | 1.08 | 0.35 |
| **Residence** |  |  |  |  |  |  |  |  |  |
| Urban (Ref.) | 1.00 |  |  |  |  | 1.00 |  |  |  |
| Rural | **1.28** | **1.13** | **1.46** | **< 0.01** |  | **1.16** | **1.05** | **1.28** | **< 0.01** |
| **Ethnicity** |  |  |  |  |  |  |  |  |  |
| Han (Ref.) | 1.00 |  |  |  |  | 1.00 |  |  |  |
| Other | **1.32** | **1.09** | **1.59** | **0.01** |  | **0.63** | **0.55** | **0.73** | **< 0.01** |
| **Parity** |  |  |  |  |  |  |  |  |  |
| 1 (Ref.) | 1.00 |  |  |  |  | 1.00 |  |  |  |
| ≥2 | **1.21** | **1.04** | **1.39** | **0.01** |  | **1.16** | **1.04** | **1.30** | **0.01** |
| **Household income quartiles** |  |  |  |  |  |  |  |  |  |
| Quartile 1 (Ref.) | 1.00 |  |  |  |  | 1.00 |  |  |  |
| Quartile 2 | 0.95 | 0.81 | 1.11 | 0.51 |  | 1.05 | 0.94 | 1.19 | 0.38 |
| Quartile 3 | 0.91 | 0.77 | 1.08 | 0.28 |  | 0.90 | 0.79 | 1.03 | 0.13 |
| Quartile 4 | 0.96 | 0.80 | 1.15 | 0.64 |  | 0.97 | 0.84 | 1.12 | 0.69 |
| Healthcare-based characteristics | | | | | | | | | |
| **Frequency of antenatal visit** |  |  |  |  |  |  |  |  |  |
| ＜5 (Ref.) | 1.00 |  |  |  |  | 1.00 |  |  |  |
| 5~7 | 1.07 | 0.92 | 1.23 | 0.37 |  | **0.75** | **0.67** | **0.83** | **< 0.01** |
| ≥8 | **1.28** | **1.09** | **1.50** | **< 0.01** |  | **0.49** | **0.44** | **0.56** | **< 0.01** |
| **Mode of delivery** |  |  |  |  |  |  |  |  |  |
| Vaginal delivery (Ref.) | 1.00 |  |  |  |  | 1.00 |  |  |  |
| Caesarean section | **0.72** | **0.64** | **0.81** | **< 0.01** |  | **1.13** | **1.03** | **1.24** | **0.01** |
| **Frequency of postnatal visit in the first 42 days** |  |  |  |  |  |  |  |  |  |
| 0 (Ref.) | 1.00 |  |  |  |  | 1.00 |  |  |  |
| ≥1 | 1.12 | 0.99 | 1.26 | 0.07 |  | 0.95 | 0.87 | 1.05 | 0.33 |
| Infant-based characteristics | | | | | | | | | |
| **Sex** |  |  |  |  |  |  |  |  |  |
| Male (Ref.) | 1.00 |  |  |  |  | 1.00 |  |  |  |
| Female | 0.97 | 0.87 | 1.09 | 0.63 |  | 1.06 | 0.97 | 1.15 | 0.22 |
| **Birth weight (g)** |  |  |  |  |  |  |  |  |  |
| ＜2500 (Ref.) | 1.00 |  |  |  |  | 1.00 |  |  |  |
| 2500~4000 | **1.98** | **1.57** | **2.49** | **< 0.01** |  | 1.16 | 0.92 | 1.45 | 0.21 |
| ＞4000 | **2.15** | **1.54** | **3.01** | **< 0.01** |  | 1.18 | 0.88 | 1.57 | 0.26 |

Abbreviations: OR-odds ratio; CI-confidence interval.
